# Genomic Informed Breeding Strategies for Strawberry Yield and Fruit Quality Traits

**DOI:** 10.3389/fpls.2021.724847

**Published:** 2021-10-05

**Authors:** Helen M. Cockerton, Amanda Karlström, Abigail W. Johnson, Bo Li, Eleftheria Stavridou, Katie J. Hopson, Adam B. Whitehouse, Richard J. Harrison

**Affiliations:** ^1^Genetics, Genomics and Breeding, NIAB EMR, East Malling, United Kingdom; ^2^University of Kent, Canterbury, United Kingdom; ^3^Cambridge Crop Research, NIAB, Cambridge, United Kingdom

**Keywords:** organoleptic, flavour, acidity, achene, QTL mapping, breeding, yield, genomic prediction

## Abstract

Over the last two centuries, breeders have drastically modified the fruit quality of strawberries through artificial selection. However, there remains significant variation in quality across germplasm with scope for further improvements to be made. We reported extensive phenotyping of fruit quality and yield traits in a multi-parental strawberry population to allow genomic prediction and quantitative trait nucleotide (QTN) identification, thereby enabling the description of genetic architecture to inform the efficacy of implementing advanced breeding strategies. A negative relationship (*r* = −0.21) between total soluble sugar content and class one yield was identified, indicating a trade-off between these two essential traits. This result highlighted an established dilemma for strawberry breeders and a need to uncouple the relationship, particularly under June-bearing, protected production systems comparable to this study. A large effect of quantitative trait nucleotide was associated with perceived acidity and pH whereas multiple loci were associated with firmness. Therefore, we recommended the implementation of both marker assisted selection (MAS) and genomic prediction to capture the observed variation respectively. Furthermore, we identified a large effect locus associated with a 10% increase in the number of class one fruit and a further 10 QTN which, when combined, are associated with a 27% increase in the number of marketable strawberries. Ultimately, our results suggested that the best method to improve strawberry yield is through selecting parental lines based upon the number of marketable fruits produced per plant. Not only were strawberry number metrics less influenced by environmental fluctuations, but they had a larger additive genetic component when compared with mass traits. As such, selecting using “number” traits should lead to faster genetic gain.

## Background

Wild strawberry fruits have evolved to attract frugivorous animals. The sweet flesh provides nutrition in return for endozoochory or the dispersal of seeds (Beal, 1898). Achenes, the true fruits, are distributed around the pseudo fruit or receptacle of a strawberry, which ensures that partial eating of a berry is likely to result in the ingestion of seeds. Digestion of seeds is required for the

“activation” of germination potential and, therefore, completion of the natural strawberry life cycle (McAtee, 1947; Nakamura, 1972; Vazačová and Münzbergová, 2013). The mutualism between birds or mammals and strawberries has led to natural selection for seed-disperser “desired” fruit quality traits. Indeed, the change in colour that develops upon ripening can act as a visual signal that ripe fruit contain seeds ready for dispersal (Schaefer et al., 2006) and some volatile organic compounds have been implicated as attractants (Ménager et al., 2004; Du et al., 2011; Rodríguez et al., 2013). Thus, wild strawberries have been naturally selected to attract dispersers. By contrast, breeders aim to artificially select strawberries to possess “human-desirable” fruit quality traits with the ultimate aim of increasing consumer consumption.

*Fragaria* × *ananassa* became the dominant cultivated strawberry species in the eighteenth century and systematic breeding was subsequently implemented to improve the fruit size and vigour of strawberry plants (Hummer and Hancock, 2009). In more recent history, strawberry breeders have succeeded in improving strawberry marketable yield and to a lesser extent fruit quality (Zorrilla-Fontanesi et al., 2011; Klee and Tieman, 2018). Indeed, fruit quality is a complex trait that is made up of multiple visual (uniformity, colour), organoleptic (flavour, texture), and sensory (firmness) factors (Cardello, 1995). Nonetheless, poor fruit quality can lead to the rejection of high-yielding cultivars by grower consortia and consumers (Brennan and Graham, 2009). Thus, improving strawberry fruit quality is a complex undertaking. Flavour is a key component of fruit quality, which requires a balance of sugar and acid, with a high total soluble sugars to titratable acid ratio believed to represent a better tasting fruit for the UK market (Mitcham, 1996; Ménager et al., 2004; Ikegaya et al., 2019). However, multiple other factors have been found to significantly impact flavour (Schwieterman et al., 2014), including the secondary metabolites associated with a peach flavour (γ-decalactone; Chambers et al., 2014) and burnt caramel flavour (mesifuran) (Sanz et al., 1995). Beyond this, there are many pleasant aroma compounds present in *Fragaria chiloensis* and *Fragaria virginiana*, the wild progenitors of strawberry. It is believed the volatiles have been lost during the breeding process and that introgression could offer an additional source of flavour (Ulrich et al., 2007).

Despite extensive strawberry improvement over recent centuries, there remains a large variation in strawberry fruit quality and consistency, both within and between cultivars due to influences of environmental factors (Schwieterman et al., 2014; Lado et al., 2019). Robust phenotyping protocols will allow accurate selection to capture this variation, thus maximising genetic gain and improving desirable traits. Organoleptic traits are complex and are predominantly assessed through subjective means, nonetheless, robust protocols have been established (Lawless and Heymann, 2013). Scientific sensorial evaluation can be undertaken by tasting panels who are trained to detect the presence and magnitude of aromas, textures, and flavours (Nakamura, 1972). However, the costs associated with such an organoleptic analysis are prohibitive for pre-breeding and early-stage selection purposes (Migicovsky, 2020). Furthermore, such tests have limited application in breeding as they do not indicate whether a trait is desirable; for which, the preference of a trait must be assessed by a consumer panel.

The ultimate aim of breeding is to produce varieties yielding fruit that achieve an enjoyable multi-sensorial eating experience leading to repeated consumer purchasing. Initial purchases have been shown to be based on appearance, however, flavour and quality were indicative of repeat purchasing (Diehl et al., 2013). Indeed, the most influential factors on US consumer purchases have been rated as taste and produce freshness (Ruth and Rumble, 2016) with strawberry sweetness and complex flavours as the most highly prized attributes, whereas nutritional content was not valued (Colquhoun et al., 2012). These complexities make fruit quality hard to dissect and lead breeding to be classified as more of an art than a science.

Molecular breeding is considered to be an effective strategy to select for traits that are expensive or difficult to phenotype. Marker assisted selection (MAS) can improve traits that are controlled by a small number of major effect genes (Wang et al., 2018). By contrast, genomic prediction can abbreviate the period associated with fixing polygenic traits of complex inheritance. Genomic prediction requires two phases—first, the training phase and second, the validation/selection phase (Karlström et al., 2019). Genomic prediction results in the generation of genomic estimated breeding values which assist the early identification of good parental lines and progeny lines allowing rapid generation cycling, and a reduction of the breeding cycle time. A reduced breeding cycle time results in a faster genetic gain, thus, creating a competitive advantage for breeding companies. Genomic selection approaches have revolutionised animal breeding, to great success (Hayes et al., 2009; Wolc et al., 2011; Swan et al., 2012; Cleveland and Hickey, 2013). The efficacy of genomic selection in strawberries has already been established, with a selection efficiency of 74% observed in increasing average fruit weight (Gezan et al., 2017). Balancing the costs of genotyping with the potential benefits of rapid genetic gain is a critical balance for plant breeders. The work outlined in this study illustrates which traits may be improved through adopting genetic breeding strategies.

In this current research, we studied a multi-parental population of strawberries to assess the phenotypic relationships between fruit traits. We assessed the potential to improve each trait and the level of variation present within the population and finally, we reported the presence of quantitative trait nucleotides (QTN) associated with traits and determine the potential efficacy of genomic selection breeding approaches. In this manuscript we present a comprehensive analysis of the genetic components influencing fruit quality and yield traits in strawberries and discuss how our findings may help to optimise strawberry breeding through the implementation of genomic approaches. In this study, we asked (1) to what extent can we parameterize and standardise sensory fruit quality assessment, (2) can robust measures truly act as a surrogate for a human scoring system, and (3) can we implement advanced breeding strategies using subjective data sets in a fashion able to assist breeding for fruit quality?

## Materials and Methods

### Plant Material and Experimental Set-Up

The multi-parental strawberry population used in this study was designed to segregate for multiple fruit quality traits. Interrelated crosses between 26 parental lines were made, to produce 26 families of between 6 and 15 individuals (average 10.8). The number of genotypes per family is denoted in Supplementary Table 1. Parental and grandparental lines were included in the population where possible. Parental cultivars included “Malling Centenary” and “Vibrant” alongside elite and low-quality accessions selected to represent diverse fruit quality traits (Refer to Li et al., 2020 for a network diagram of family relationships). A total of 270 genotypes and 28 progenitors were assessed in this study. Plants were raised and allowed to go dormant over the autumn and early winter before being placed in a −2°C cold store. After 5 months, one cold-stored strawberry plant per genotype was potted up into coir and grown under ambient polytunnel conditions. Subsequent replicate plants of each genotype were removed from the cold store at 3-week intervals, with each cohort of plants forming a replicate block. Five replicate blocks of plants were set up along table-top gutters within covered polytunnels. Each block was treated with a standard pest and disease pesticide regime throughout the season. Ambient environmental conditions during the first 40 days of fruit development were: block one 17.8 ± 4.5°C, 79.6 ± 4.5% RH; block two 20.3 ± 4.9°C, 72.4 ± 20% RH; block three 20.3 ± 5.1°C, 74.2 ± 20.2% RH; block four 18.6 ± 4.9°C, 81.27 ± 17.7% RH; block five 17.3 ± 4.5°C, 83.3 ± 16.5% RH. Fruit from block one was not consumed due to the harvest interval associated with the application of a plant protective chemical, all other traits were assessed for block one. The experiment was situated at East Malling (NIAB EMR), Kent, UK (51° 17′ 24.202″ N 0° 26′50.918″ E) along two 150 m long polytunnels covered in 150-micron plastic covers. Even pollination was assisted through the addition of a Natupol Koppert bumble beehive into each tunnel (Koppert Biological Systems, Berkel en Rodenrijs, Netherlands). Plants were grown in coir in 2 L pots, and fertigation was supplied at 1 kg Vitax Vitafeed (Vitax Limited, Leicester, UK) (N:P:K, 176:36:255) L^−1^ (10 s^−1^ 45 m). Replicated blocks represented both planting date and tunnel position. The picking date varied for each berry as strawberries were picked when ripe between 11 July 2018 and 8 November 2018. The fruits were picked every weekday and assessed on the day of picking. Fruit quality traits were assessed using three berries where possible for each replicate plant across the five blocks. Yield metrics were assessed on every pick and later summed to provide a total end-of-season value for assessment.

### Phenotyping

**Figure 1:**
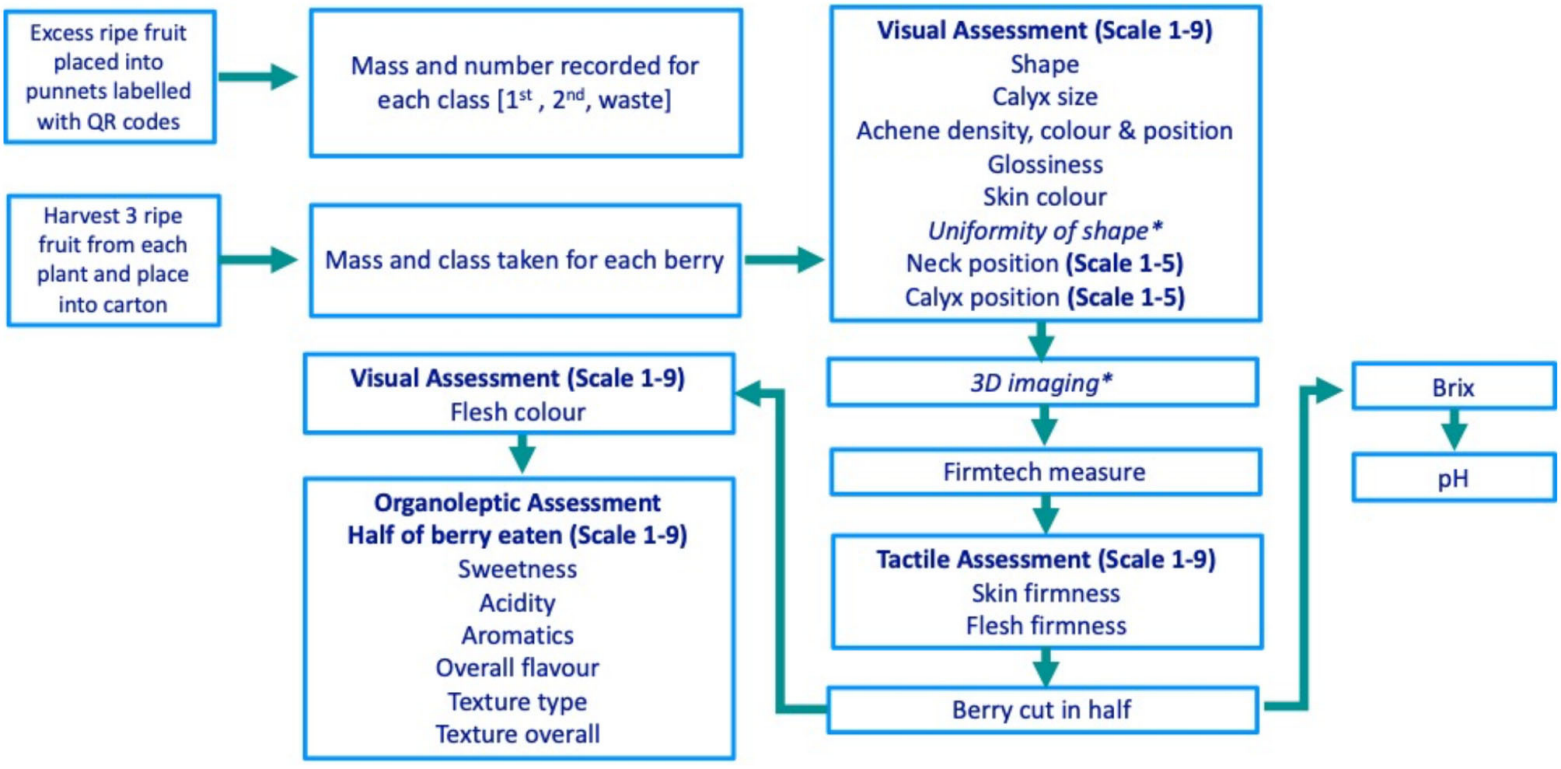
The strawberry phenotyping process from the picking of strawberries through to destructive assessments. Each box represents a discrete phenotyping station ^*^Uniformity of shape and 3D imaging have been reported by Li et al. (2020).

The phenotyping process is detailed in Figure 1. Ripe fruits were harvested into individual punnets for each genotype, and berries were then classified based on size and quality (class one; 28–45 mm diameter, class two; <28 mm diameter and waste; either misshapen/physiological/pathological damage) and the number and mass of berries per plant and per class were recorded. Primary and secondary ripe strawberries (as defined by Savini et al., 2005) were hand-selected into segmented cartons before measurement. Punnets and cartons were labelled with QR codes to allow data entry using the Field Book app (Rife and Poland, 2014). Visual, tactile, and organoleptic strawberry traits were scored on a 9- or 5-point scale (Figure 1), with score standardisation training provided for all assessors. Trait assessment descriptors, alongside the nine discrete categorical shape and texture categories, can be found in Supplementary Table 2. Traits were rated for importance in breeding on a scale from 1 (not important) to 9 (highly important) as defined by breeders at NIAB EMR. Three-dimensional imaging was conducted as outlined in the study of Li et al. (2020), wherein the height to width ratio (H:W) was calculated using 3D berry images and used to represent strawberry shape. Firmness measures were taken using a FirmTech FT7 machine (UP GmbH, Ibbenbüren, Germany). Berries were cut longitudinally to allow half of the berry to be assessed for organoleptic properties by one of four assessors. Total soluble sugars and pH were measured from juice squeezed from the remaining half of the berry using a refractometer meter (Atago PAL, ATAGO®, Tokyo, Japan) and pH meter (LAQUA twin B-712, HORIBA Scientific, Palaiseau France), respectively. Halved strawberry samples did not provide sufficient juice to measure titratable acidity.

### Genotyping and Linkage Map

Deoxyribonucleic acid was extracted from the population using the Qiagen DNeasy plant mini extraction kit (Qiagen, Düsseldorf, Germany). The Axiom® IStraw35 384HT array (i35k) (Thermo Fisher Scientific, Waltham, MA, United States) was used for genotyping (Verma et al., 2017a). The i35k array is based on a streamlined set of informative single nucleotide polymorphisms (SNP) first developed for the 90k Affymetrix Axiom® array. The 90k array was developed through genome sequencing and SNP discovery in 19 octoploid cultivars and six diploid strawberry species (Bassil et al., 2015). The NIAB EMR strawberry consensus map was used to define marker positions (Cockerton et al., 2018). *Fragaria* × *ananassa* chromosome number is denoted by 1–7 and the sub-genome number is represented by A–D as specified in the study by van Dijk et al. (2014).

### Statistical Analysis

The best linear unbiased estimates (BLUE) were calculated for each genotype and trait using a linear mixed effect model that included the cofactors of the assessor, individual, picking date, and block. The model type fitted was specified individually for each trait as detailed in Supplementary Table 3. Significant covariates were identified through comparison of a mixed model (phenotype ~ genotype + block + individual + date + assessor) to a model omitting the trait of interest, comparisons were made using a likelihood ratio test. Significant genotype × environment (G × E) interactions were assessed as specified for co-factors above but with the inclusion of the date of picking × genotype interaction variable. A principal component analysis (PCA) was conducted on a “scaled” phenotypic correlation matrix using the core R package “stats.” Heritability values were calculated using the R package “heritability” (Kruijer et al., 2016) where broad-sense heritability is H^2^ = σG2/(σG2 +σE2/r) was calculated based on analysis of variance statistics, where r is replicate number, G represents genotypic variance, and E represents residual error. Narrow-sense heritability was calculated by h^2^ = σA^2^/ (σA^2^ +σE^2^), where A represents additive genetic variance, where the relationship matrix was calculated using the R package “snpReady” (Granato and Fritsche-Neto, 2018). Phenotypic correlations were calculated using the R package “psych” (Revelle, 2017) and plotted using the R package “corrplot” (Wei and Simko, 2017), *p*-values were adjusted for multiple testing.

### Genomic Analysis

**Figure 2:**
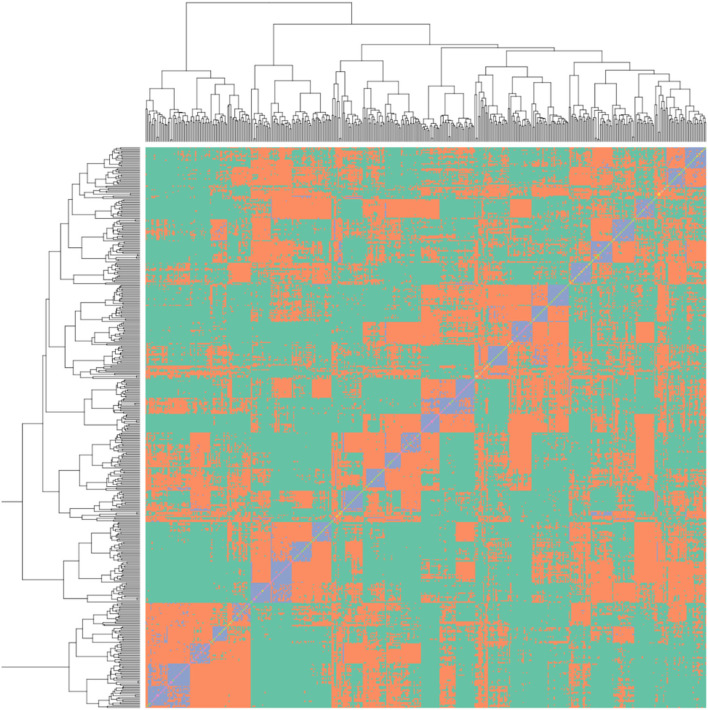
Genetic relationship matrix for the strawberry multi-parental population, blue colouring represents the full sibling relationships, orange represents half-sibling relationships between individuals, green represents less than half-sibling relationships. The relationship within the 26 families can be observed in the blue squares along the diagonal.

The R package “snpReady” was used to generate a genetic relationship matrix (Figure 2) and the R package “rrBLUP” was used to conduct a genome-wide association study (GWAS) analysis (Endelman, 2011). The rrBLUP model was y = Zg + Sτ + ε, where y is phenotypic observations, Z and S are matrices of 0s and 1s representing the fixed effects of; β the population structure, g the genetic background and τ the additive SNPs (Yu et al., 2006). GWAS was conducted with the genetic relationship covariance matrix added as a random effect and a minor allele frequency set to 5%. A Bonferroni corrected *p-*value of 0.001 was used to identify significant QTN. The values for the *R*^2^ of QTN effect size were calculated using a linear model comparing BLUE calculated values vs. predicted values assuming an additive relationship between focal SNPs. A genomic best linear unbiased prediction (GBLUP) was calculated using the software ASReml-R (Gilmour et al., 2015). A five-fold random subdivision of the population into the “training” (80%) and “test” (20%) was used as suggested in the study by Erbe et al. (2010). The genomic selection GBLUP linear mixed model specified a variance structure that combined genotype and the inverse genetic relationship matrix as random variables. Predictive ability was defined by the correlation between the predicted and BLUE score for the test population over 100 permutations with a random selection of the genotypes forming the “test” and “training” population, thus allowing us to determine the predictive ability of the model. Prediction accuracy was calculated as detailed in the study of Gezan et al. (2017).

## Results

### Variation in Fruit Quality and Yield

A total of 19 strawberry fruit quality and 12 yield traits were measured as part of the fruit phenotyping platform (Supplementary Table 3). Fruits from 270 genotypes were assessed in five separate plantings replicated across the season. All measured traits were found to have significant genetic and environmental components (Supplementary Table 3). Comparison of full mixed models with models omitting the test covariate indicated that the date of picking and block significantly influenced all traits. However, variation in block was superseded by variation in picking date for the following traits: flesh colour, acidity perception, sweetness perception, pH, and flavour perception. When assigned as a factor, the assessor was found to influence the scores for multiple traits, however, interestingly the assessor did not significantly influence the scores of skin colour, acidity perception, achene density, achene colour, and flesh firmness (Supplementary Table 3). Significant G × E terms indicate that different genotypes do not produce a consistent response across environments. The variation and magnitude of fruit quality phenotypic scores for each trait, both within and between families and blocks can be seen in Supplementary Tables 1, 4, respectively.

**Figure 3:**
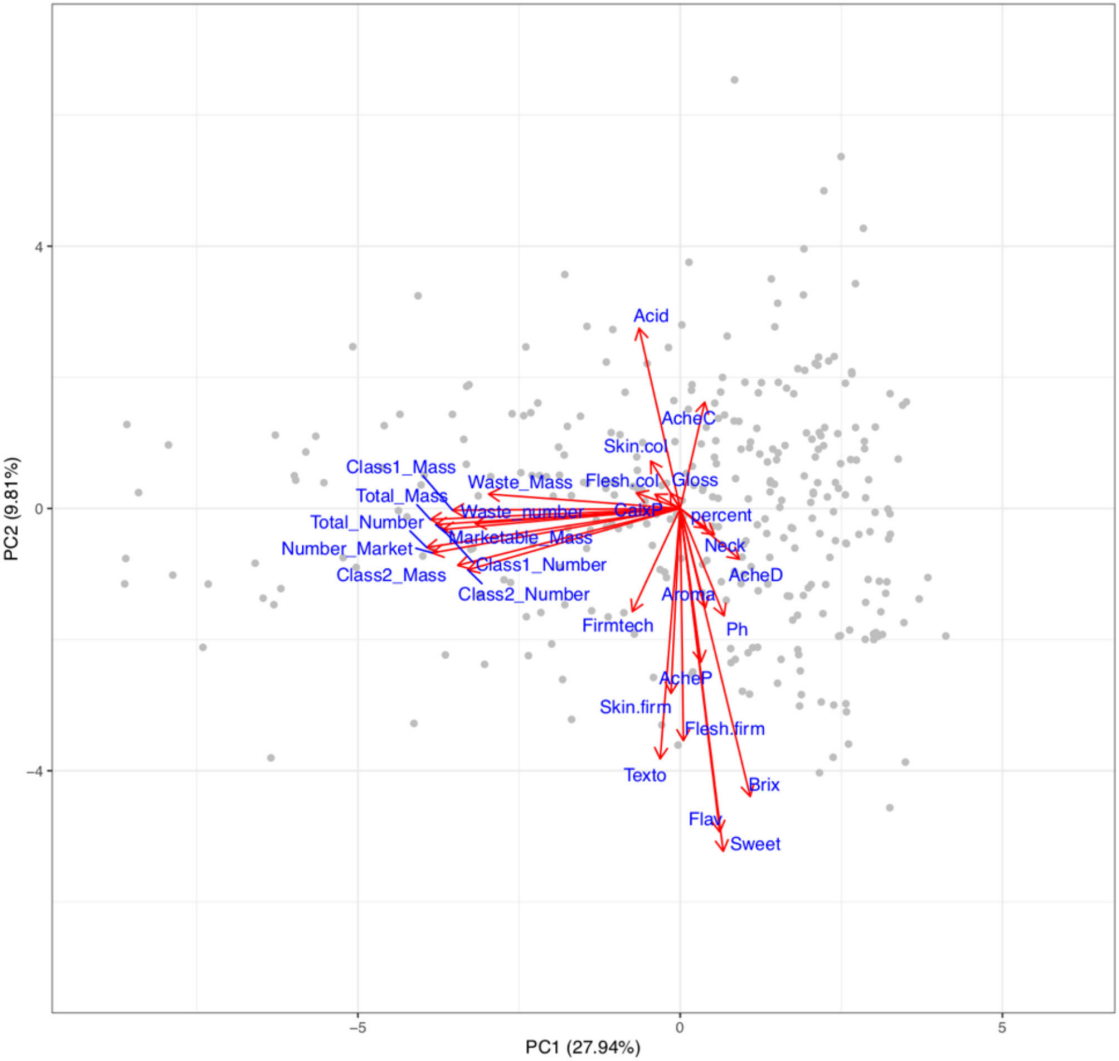
Biplot representing variation in fruit quality and yield traits within the multi-parental strawberry population. Numbers in brackets represent the proportion of variation explained by principal components (PC). Red arrows indicate the relative influence a trait has on the PC each associated with the trait denoted by a blue label. Grey points represent genotypes. CalxP, calyx position; Skin.col, skin colour; Flesh.col, Flesh colour. Acid, acidity perception; AcheC, Achene colour; Neck, Neck position; Shape, height:width; Texto, texture rating overall, Fimtech, automated firmness; Flesh. Firm, flesh firmness manual; Skin.firm, skin firmness; Gloss, Glossiness; percent, percentage of marketable fruit; AcheP, Achene position; AcheD, Achene density; Ph, pH; Aroma, aromatic strength perception; Brix, total soluble sugars; Flav, Flavour; Sweet, Sweetness perception.

**Figure 4:**
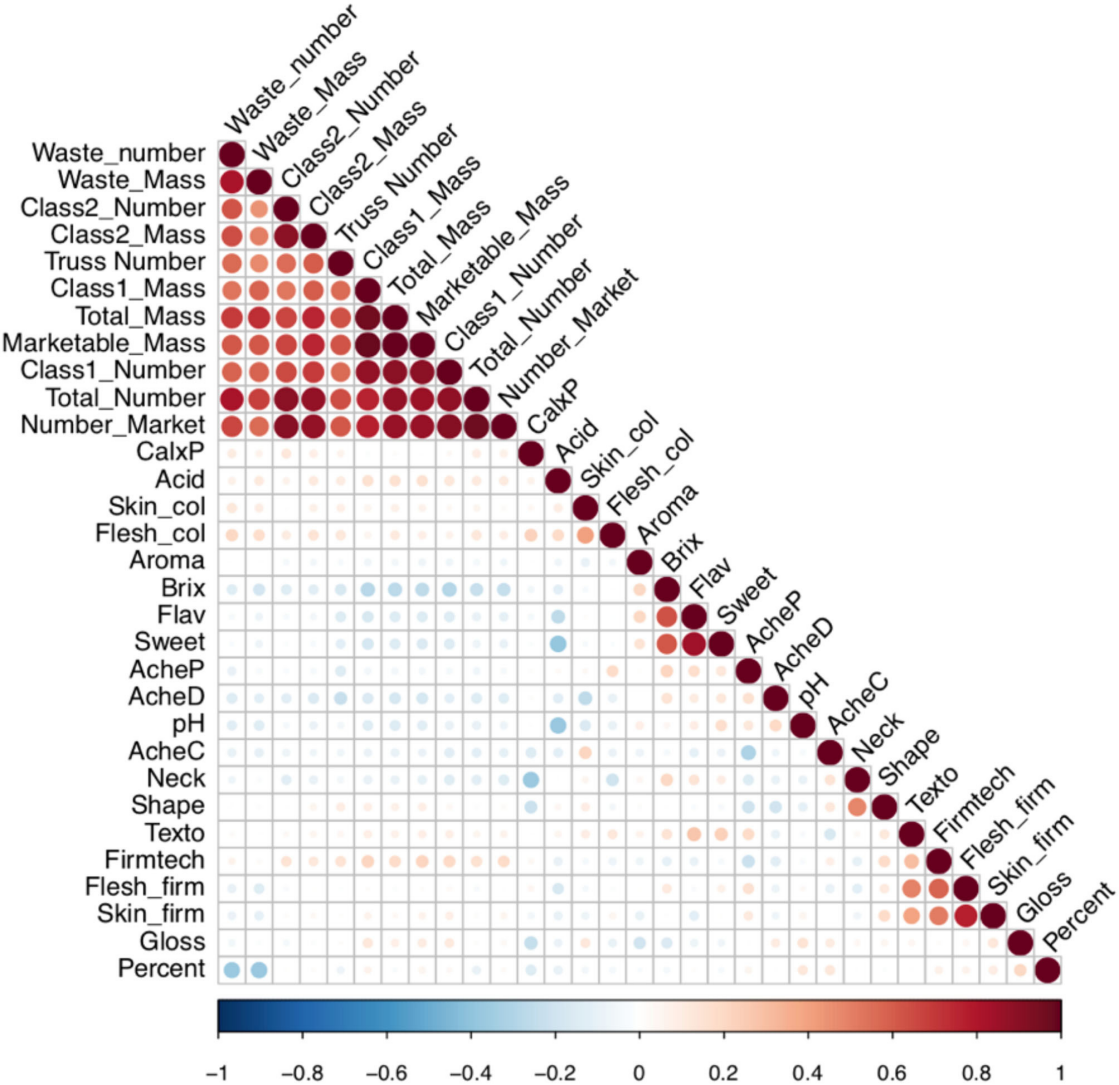
Correlation matrix between the fruit quality and yield traits within the multi-parental strawberry population. The strength of colour denotes the magnitude and direction of the correlation coefficient. The size of the circle denotes the significance value. CalxP, calyx position; Skin_col, skin colour; Flesh_col, flesh colour. Acid, acid perception; AcheC, achene colour; Neck, neck position; Shape, height:width; Texto, texture rating overall; Fimtech, Firmness, instrument; Flesh_firm, flesh firmness manual; Skin.firm, skin firmness; Gloss, glossiness; Percent, percentage of marketable fruit; AcheP, achene position; AcheD, achene density; Aroma, aromatics; Brix, total soluble sugars; Flav, flavour perception; Sweet, sweetness perception.

The power to alter traits generally depends upon the presence of the variation within the breeding germplasm. Therefore, visualisation of variation is required to define the boundaries within which traits may be improved. The relationship between the fruit quality and yield traits within the multi-parental population is depicted in a PCA biplot and correlation matrix (Figures 3, 4). Results from the PCA showed that PC1 accounted for 27.9% of the variation and was largely correlated with fruit number and mass, whereas PC2 represented 9.81% of the variation and was correlated with organoleptic traits.

Broad-sense heritability values (Supplementary Table 3) show that different proportions of the variation observed in traits were controlled by genetic factors. Aroma (0.03), the mass of waste fruit (0.08), and percentage mass (0.21) had the lowest broad sense heritability values, whereas truss number (0.90), neck position of fruit (0.74), and flesh firmness (0.68) has the highest broad sense in the heritability. By contrast, narrow-sense heritability scores show that between 0 and 45% of the variation was due to additive genetic effects (Supplementary Table 3).

### QTN Discovery and Genomic Prediction

A total of 108 unique QTN were detected across 10 of the 19 fruit quality and 7 of the 12 yield traits measured (Supplementary Table 5). Among the identified markers, 23 were associated with yield traits, 17 of these were found to be associated with “number” traits. Whereas six markers were associated with “mass” traits, of which, only one was not identified using the number yield metrics.

A total of 85 unique QTN were found to be associated with fruit quality traits, the most important traits will be detailed in the dedicated sections below. Many of the ancillary traits were found to be associated with QTN. In this study, 19, 12, and 3 alleles were found to represent internal flesh colour, neck position, and calyx position, respectively. Achene position was associated with eight QTN, achene density with three QTN, and achene colour with two QTN (Supplementary Table 3). However, no QTN was found for many of the subjective traits: aroma, sweetness perception, overall rating of texture, skin colour, flavour, and glossiness. Similarly, no QTN was found for several objective traits: soluble sugar content, objective firmness, and truss number. The correction threshold was very stringent, thus eliminating the possibility of false-positive QTN (Supplementary Table 3).

Comparison of phenotypic values with those predicted using a GBLUP model provides an indication of which traits may be improved through a genomic selection breeding approach. The highest prediction accuracy values were seen for flesh firmness (0.54) and neck position (0.49), whereas the lowest values were observed for aromatics (−0.02) and glossiness (0.13) (Supplementary Table 3). Furthermore, the traits of skin colour, overall texture rating, and truss number, for which we did not identify any QTN, had prediction accuracy values between 0.32 and 0.34 (Supplementary Table 3). A wealth of results has been generated due to the large number of phenotypes assessed in this study, here we seek to highlight the notable results relating to the traits rated as important by breeders.

### Fruit Yield and Class

There was a strong positive correlation between the total number of fruits, number of marketable fruits, and number of class one fruits with marketable fruit mass (*p* < 0.00001; Figure 4). However, a negative relationship was observed between total soluble sugars and class 1 yield metrics indicating that high-yielding June-bearing varieties were associated with reduced sugar content (*p* < 0.05, *r* = −0.22).

There were 14 QTN associated with total fruit number. Together these had an *R*^2^ of 0.31 indicating that almost a third of the variation can be explained by the identified SNPs (Supplementary Table 3). The magnitude of variation in yield can be viewed in Supplementary Table 6. Many of the yield QTN were identified multiple times through association with the different traits and were represented by the same significant SNP. In total, 17 of the progenitors were homozygous for the favourable SNP, while the remaining progenitors carried a single copy of the favourable allele. This illustrates that the SNP is abundant in the germplasm studied and could be targeted through MAS to improve the quantity of high-class fruit.

### Sweetness and Acidity

**Figure 5:**
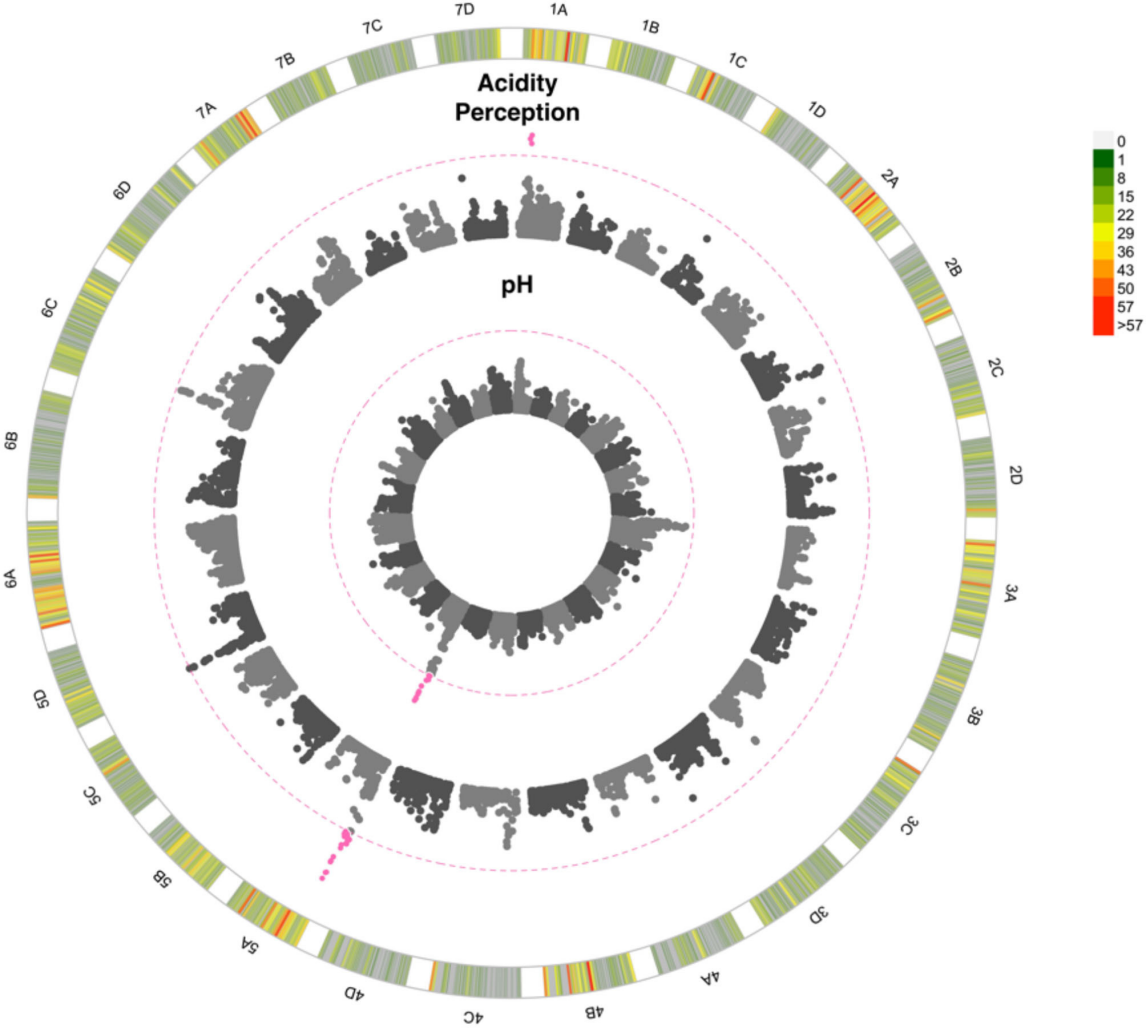
Manhattan plot of GWAS looking at the association between SNPs and strawberry acidity. 1A to 7D represent the 28 chromosomes of the strawberry genome. The inner Manhattan plot represents acidity perception, the outer plot represents pH. The pink dotted line represents Bonferroni correction at –log10 *p* = 7.14 pink points are those which pass the significance threshold. Marker positions are scaled to the *Fragaria vesca* genome v.4 (Li et al., 2019). The colour-coded key in the outermost circle represents the number of SNPs segregating at each point across the chromosome.

Flavour, sweetness perception and total soluble sugars were all shown to be positively correlated (*p* < 0.00001; *r* > 0.6; Figure 4). Both sweetness perception (*p* < 0.00001; *r* = −0.38), and to a lesser extent flavour (*p* < 0.001; *r* = −0.28), were negatively correlated with acidity perception, indicating acidity may impair good flavour. Total soluble sugar values varied between 3.8 and 17.9 °Bx with an average of 9.37 °Bx (Supplementary Table 1). No QTN was detected for sweetness perception nor soluble sugar content. By contrast, a highly significant QTN was detected on chromosome 5A for acidity perception and pH measurements (Figure 5). This QTN was represented by the same significant marker or focal SNP (Supplementary Table 5). Detection of the QTN was greater for the subjective trait of acidity perception and there was no significant effect of the assessor. A second QTN was associated with acidity perception on chromosome 1A, this was not detected through pH measures. Prediction ability values of 0.4, 0.29, 0.35, and 0.21 were found for pH, acidity perception, total soluble sugars, and sweetness perception, respectively.

### Texture, Skin Firmness, and Fruit Firmness

**Figure 6:**
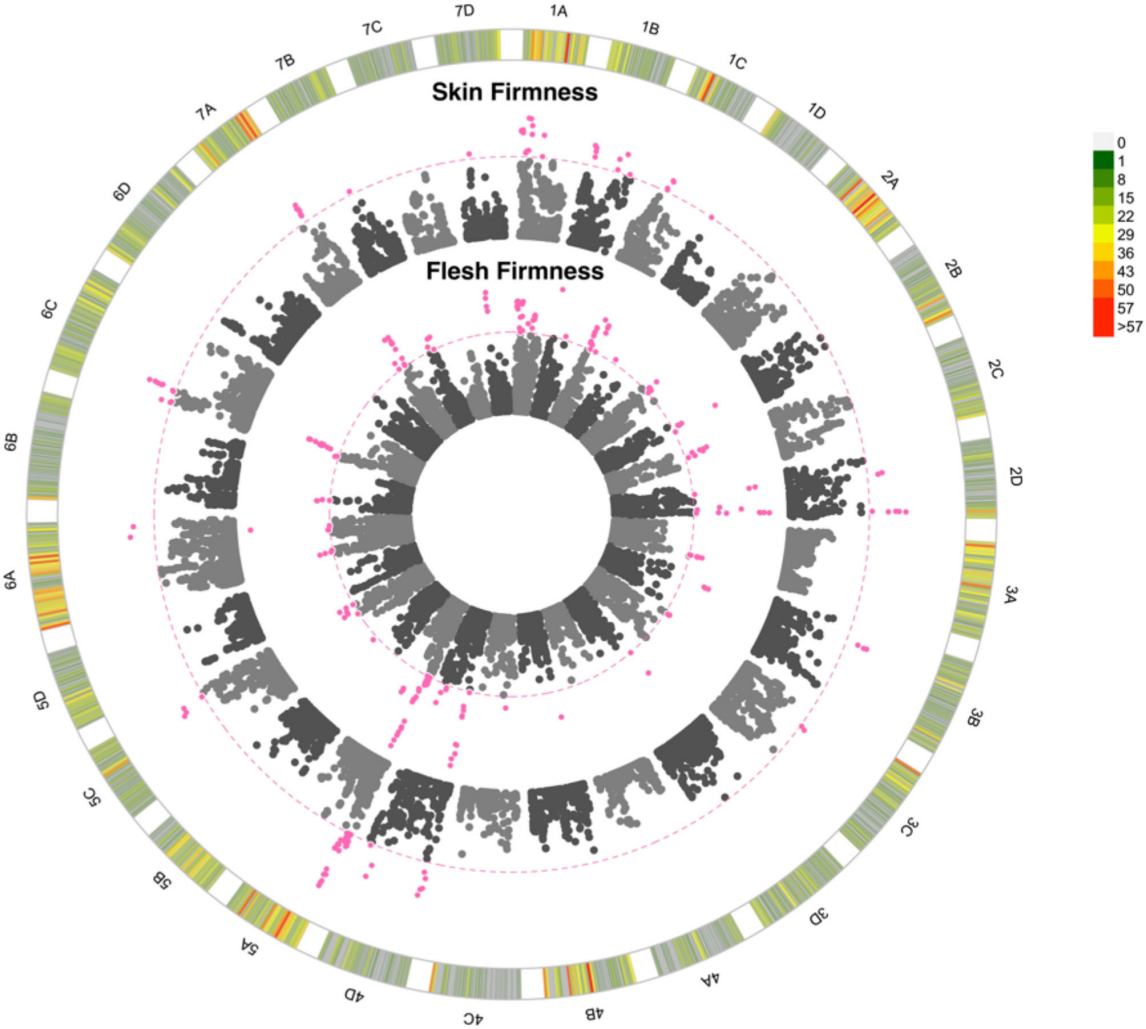
Manhattan plot of GWAS looking at the association between SNPs and strawberry fruit firmness. 1A to 7D represent the 28 chromosomes of the strawberry genome. The inner Manhattan plot represents flesh firmness, the outer plot represents skin firmness. The pink dotted line represents Bonferroni correction at –log10 *p* = 7.14, pink points are those which pass the significance threshold. Marker positions are scaled to the *Fragaria vesca* genome v.4 (Li et al., 2019). The colour-coded key in the outermost circle represents the number of SNPs segregating at each point across the chromosome.

Skin firmness scores varied from very fragile-fragile (2) to very strong (9; Supplementary Table 1) and flesh firmness scores varied between very soft-soft (2) and very firm (9; Supplementary Table 1). Both genotype and environment significantly influenced skin and flesh firmness, with the assessor impacting the score of skin firmness but not flesh firmness. Skin firmness, flesh firmness, automated firmness, and texture ratings were all positively correlated (*p* < 0.00001; *r* > 0.29). A total of 24 and 15 QTN were found to represent flesh firmness and skin firmness, respectively. The *R*^2^ values of 0.33 and 0.31 were associated with the presence of the QTN for flesh firmness and skin firmness respectively, these illustrate that a moderate proportion of the variation observed can be explained by the identified QTN. These QTN are particularly notable since both firmness traits are rated as 8 out of 9 for importance. Many of the skin and flesh firmness QTN co-localise, with four of the shared QTN improving both traits simultaneously whereas two QTN impact upon the traits antagonistically (Figure 6). The GBLUP model for flesh firmness has a predictive accuracy of 0.54, whereas the model for skin firmness has a predictive accuracy of 0.46 (Supplementary Table 3). The *R*^2^ illustrates the proportion of variation explained by the identified QTN; the *R*^2^ values for firmness traits were both greater than 40%, indicating a large proportion of variation that can be explained by the identified QTN (Supplementary Table 3). By contrast, automated firmness measures (although positively correlated with other firmness measures) did not reveal any QTN. Firmness is not only important for longevity, but also related to strawberry texture in a nonlinear fashion; in this study, texture type was recorded alongside the texture rating, and we see that texture types from across the firmness spectrum score low texture ratings, i.e., “woolly,” “slimy,” “stringy,” and “too crunchy” (Supplementary Figure 2).

### Fruit Shape

Since the shape is an ordinal trait, a quantitative measure of strawberry shape was adopted by measuring the H:W of each berry. H:W ratio is a continuous trait that allows data from across the population to be used in genetic analysis. Three QTN were associated with the H:W ratio on chromosomes 3A, 5B, and 6A, the alleles combined explained a total of 16% of the variation (Supplementary Tables 3, 5). The prediction accuracy of this trait was 0.4 (Supplementary Table 3). Nonetheless, H:W could not distinguish between “desirable” and “undesirable” strawberry shapes as defined by breeders at NIAB EMR (Supplementary Figure 3).

### Genomic Informed Breeding

**Figure 7:**
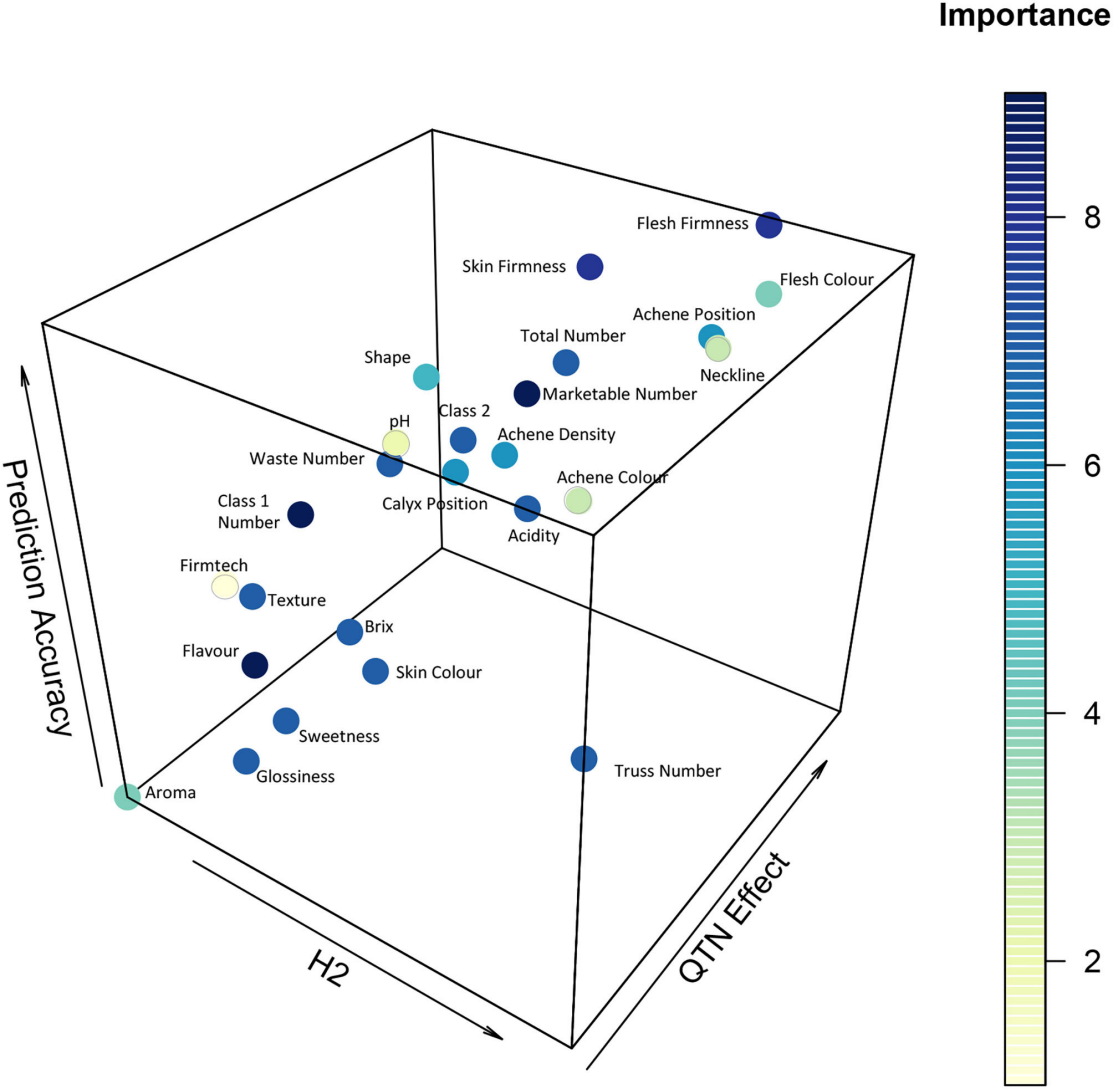
Heritability (H2), QTN Effect is the total *R*^2^ of identified alleles and prediction accuracy for strawberry yield and fruit quality traits as assessed across the multi-parental population. Dark blue represents the most important traits to select upon, yellow the least important traits.

Through plotting the importance of a trait as defined through breeding priorities against heritability, predictive accuracy, and number of QTN on a 3D scatter plot, it was possible to visualise the relative ability vs. the desire to improve yield and fruit quality traits within the study population (Figure 7). The figure provides an indication of whether the observed variation is highly heritable and whether it may be appropriate to adopt a genomic prediction or MAS breeding approach. Explicitly, traits possessing high QTN numbers and high prediction accuracy values, such as flesh firmness, are appropriate for selection using a genomic prediction breeding approach. By contrast, traits possessing low QTN numbers (one or two) and high heritability may be suitable for MAS, particularly where QTN effect sizes are high. When comparing yield traits, the number of marketable fruits was shown to have the greatest importance, as measured by breeding priorities, and also the greatest genetic component as measured by prediction accuracy, heritability, and QTN effect (Figure 7). These results indicate that the number of marketable fruits would be the best trait to pursue and select upon if using a genomic selection approach. By contrast, mass traits were associated with fewer QTN with the exception of class 2 mass (Supplementary Table 3). The lack of total strawberry mass QTN may be explained by the large influence of environmental factors upon the mass of berries.

## Discussion

### The Trade-Off Between Class One Yield and Soluble Sugar Content

We confirmed a well-established challenge for strawberry breeders: a negative trade-off (*r*= −0.22) was observed between total soluble sugars and class one plant yield metrics in June-bearing plants grown under a protected production system. Physiological or genetically linked trade-offs fundamentally limit the possibility that some combinations of phenotypes can occur (Weih, 2003). Ultimately, the traits are diametrically opposed, with the benefit gained by increasing the class one yield of strawberries, associated with a cost that leads to reduced sugar content in the resulting berries. Conceptually, should the mechanism be defined, gene editing offers a solution to overcome genetically linked traits. Unfortunately, physiological trade-offs represent a potential “roadblock” in the pursuit of an unattainable goal (Weih, 2003). Dividing a finite amount of sugar between a defined number of berries may be considered a physiological trade-off. However, gene editing or extensive breeding can still provide a solution; through the introduction of compounds that increase the perception of sweetness and flavour without the need for sugars (Schwieterman et al., 2014). Volatile organic compounds have a lower carbon cost and can improve strawberry flavour perception (Schwieterman et al., 2014). The introduction of these compounds into germplasm may become a critical component of mitigating the observed trade-off.

Further investigation is required to confirm the mechanism underpinning the relationship between yield and sugar content. Nonetheless, other studies of strawberries have hinted at the existence of this phenomenon, with a similar trade-off found in one out of 3 years across a biparental population (Zorrilla-Fontanesi et al., 2011) and a 27% increase in yield associated with an 8% reduction in soluble sugars (Whitaker et al., 2012). Our results indicated that breeders and strawberry plants alike may have to “decide” whether to invest in a greater number of berries or produce a smaller number of higher sugar content berries, with the elected strategy influencing both commercial successes for the breeder and reproductive success for the plant.

### Increasing Class One Yield

We highlighted a commercially relevant QTN on linkage group 5A associated with a 10% increase in class one fruit number. In this study, we used a diverse multi-parental population generated from temperate European germplasm, therefore, the linkage between the trait and the associated QTN can be seen to be conserved across germplasm. Past work using very sparse linkage maps has been able to identify weak signals of a QTL controlling fruit number on several chromosomes including chromosome 5 (Zorrilla-Fontanesi et al., 2011). This QTL may be reflected in our findings, but crucially, our analysis used a large number of SNPs and has provided a fine-scale resolution of the region of interest. Dissection of the components which underlie the class one category will reveal the biologically relevant attributes believed to result in higher class one yield: fruit size or truss architecture.

### Sweetness and Acidity

The use of a multi-parental population has an advantage over biparental QTL mapping studies as it allows the assessment of genetic components across diverse germplasm. A similar analysis has been conducted across a multi-parental population in strawberries where multiple QTL were identified for titratable acidity, pH, and total soluble sugars (Verma et al., 2017b). Multiple QTL for pH had been reported from a biparental study, one of which was on chromosome 5B (Lerceteau-Köhler et al., 2012). The large effect acidity perception and pH QTN observed in our study was located on linkage group 5A, and so may represent a novel source of flavour that has not been reported in the literature previously. Another study reported a QTL on linkage group 5A, but this was a large distance (10 Mb) away from the site of our identified allele (Rey-Serra et al., 2021). Furthermore, a QTL for titratable acidity was detected on linkage group 1A, which was only 0.6 Mb away from the pH allele that we detect. It is, therefore, possible that this QTN may represent the same source of acidity (Rey-Serra et al., 2021). In this study, we found that the acidity QTN was more significant for the perception ratings compared with the pH measures and there was no significant difference in the perception score per assessor. These results indicated that acidity was perceived consistently between individuals, and thus, human perception may act as a robust descriptor for strawberry acidity (Supplementary Table 3). Others have characterised the complex relationship between soluble sugar content and sweetness perception and how perception can be influenced by volatiles (Schwieterman et al., 2014). However, less has been reported on the relationship between acidity and acidity perception and our finding suggests that the relationship could be more straightforward.

### Fruit Firmness

Firmness is an essential component of fruit quality which is linked to increased shelf life, lower mechanical injury, and reduced susceptibility to storage rots (Maas, 1978; Hietaranta and Linna, 1999). Overall, breeders aim for an intermediate level of firmness, striking a balance between durability and a desirable eating texture. There were a large number of QTN identified for fruit firmness, and these QTNs accounted for a large proportion of the variation observed in the multi-parental population. Therefore, firmness is likely to show improvement through the adoption of genomic prediction approaches.

A non-destructive, firmness measuring instrument was used to produce an objective measure of fruit firmness. However, these measures were not associated with high heritability, predictive ability nor QTN number. Such inconsistent results between methods of measuring strawberry firmness have been well documented (Døving et al., 2005), and our results highlighted the difficulty associated with objective measurement of this trait. We confirmed that tactile human perception can be used as a robust measure to assist the genetically guided improvement of skin and flesh firmness. Destructive penetrometer instruments may be more effective in capturing human perceived firmness, particularly where injury to the fruit is not prohibited due to downstream assessment requirements.

A study on fruit firmness in a multi-parental strawberry population assessed fruit firmness using a 9-point scale, similar to the one in this study. However, no QTL was found to be associated with firmness (Verma et al., 2017b). These findings are in contrast with this study, wherein 24 QTN were associated with fruit firmness which made it clear that the source of the material and the presence of variation within the population is a factor influencing genetic allele discovery. By contrast, another study that used a penetrometer, has found two firmness QTL on chromosome 7C and 1A (Rey-Serra et al., 2021). The focal SNP identified on chromosome 1A falls within the QTL region reported in the study by Rey-Serra et al. (2021), adding weight to the use of this region in marker assisted breeding. However, no QTN was identified on chromosome 7C in this study. The high number of alleles associated with firmness found in this study implies that a genomic prediction strategy should work well to incorporate the observed variation. This is also supported by a high prediction accuracy for fruit firmness (0.54). Future studies could determine whether the alleles detected in this study are associated with changes in polygalacturonase genes (Posé et al., 2013).

Limited genetic studies have been conducted on strawberry texture, and this may be due to the complexities associated with quantifying the trait. Nonetheless, texture has been reported to play a significant role in the overall fruit quality score of strawberries (Cockerton et al., 2020), therefore, the desirable texture of strawberries must continue to be selected for despite the associated challenges.

### Fruit Shape

The H:W ratio can be used to discriminate between some strawberry shape types, particularly long conic fruit. Indeed, we found three QTN associated with shape, two of which are on the same linkage group (3A and 6A) but at different locations to those identified in bi-parental populations (Rey-Serra et al., 2021). However, the H:W ratio did not segregate desirable and undesirable fruit shapes into discrete groups. The lack of a relationship represents a discord between the desirability of a given shape (as detailed in Li et al., 2020) and the biologically measurable trait H:W. As such, H:W cannot be used as a straightforward metric to select for fruit shape as the breeders' definition of desirable strawberry shape does not align with the H:W measure. However, H:W or a similar metric is needed to study the underlying genetic components associated with the trait and, thus, allow the modification of shape through genome-informed breeding. More comprehensive methods of fruit shape quantification have been conducted through the use of machine learning approaches (Feldmann et al., 2020) alongside 3D imaging studies describing fruit uniformity (Li et al., 2020). Strawberry shape has been studied extensively in the diploid strawberry *F. vesca* and the genes responsible for controlling the height and width of the berries have been identified (Wang et al., 2017; Liao et al., 2018). Plant hormones have been shown to define fruit shape in *F. vesca*, with auxin boosting the width of receptacle expansion, GA increasing height, and abscisic acid (ABA) inhibiting overall expansion (Wang et al., 2017; Liao et al., 2018). Further study may determine whether similar genetic components control the complexities of fruit shape in octoploid strawberries.

### Genomic Informed Breeding

In this current research, we studied the power to breed for traits vs. the relative importance in breeding for them. Improving yield is a key goal of plant breeding. Critically in this study, we identified 11 SNPs associated with an increase in marketable yield, including one marker that specifically enhances class one fruit, these markers can be used in marker assisted breeding. Truss number directly influenced the number of strawberries produced, this trait has a high broad-sense of heritability (90%) indicating that it is a highly heritable trait and yet a lower narrow-sense heritability (26%) with no QTN detected. These results indicated that the trait may have a highly polygenic nature or potentially involves complex epigenetic interactions. Truss number was associated with a prediction ability of 0.33 as calculated *via* additive models, indicating that there is the potential to increase truss number through genomic selection.

More importantly, our findings suggested that the number of marketable fruits per plant may be the best trait to select upon when breeding for high cropping strawberry varieties, particularly when using genomic prediction approaches. Enhancing the accuracy of selection is a critical component for enhancing genetic gain (Cobb et al., 2013). The only way that improvement can be made *via* breeding is through selecting upon the variation that is caused by genetic components. Therefore, the selection of variation that is largely influenced by environmental conditions (such as mass) will lead to lower genetic gain. It should be acknowledged that mass traits were more influenced by environmental components and had lower narrow sense heritability scores, compared with fruit number traits. As such, using mass traits for yield selection is associated with lower accuracy. Therefore, we suggest that selecting based upon the number of marketable strawberries could improve the accuracy of selection and, thus, lead to greater genetic gain. However, to prevent selection for smaller and yet marketable berries, it is recommended that breeders increase the threshold for acceptable berries.

### Environmental Influence on Fruit Quality

Homeo-QTL, whereby QTL were located at the same physical position across different subgenomes, have been identified in previous studies for fruit shape, size, glucose content, pH, malate content, and firmness traits (Lerceteau-Köhler et al., 2012). The researchers found that different QTL homologues were expressed under different environmental conditions. Therefore, it was hypothesised that, fruit quality is an important trait associated with reproductive success, and that multiple gene homologues remain functional. Environmental variation has a large impact on strawberry fruit production, indeed, some cultivars of strawberries grown under high temperatures have been shown to produce lower yields (Sun et al., 2012) and poorer flavour (Wang and Camp, 2000). Our experimental setup, whereby blocks were temporally separated across the season, prohibited homeo-QTL detection but allowed us to mitigate the significant impact of environmental variation on traits (Supplementary Table 3); thus, strengthening our the ability to detect stable alleles operational across multiple environments.

## Conclusions

Through studying the genetic composition of strawberry traits, we conclude that selecting upon the number of marketable fruits produced per plant may lead to the production of high-yielding strawberry varieties. We showed that subjective human scores of firmness and acidity perception were superior to surrogate measures of non-destructive instruments and pH meters and recommend the implementation of genomic prediction and MAS to capture the observed variation, respectively. Finally, we highlight the dilemma faced by many strawberry breeders: greater class one yield or sugar content?

## Data Availability Statement

The original contributions presented in the study are included in the article/Supplementary Materials, further inquiries can be directed to the corresponding author.

## Author Contributions

AJ, HC, BL, ES, and RH: conceived and designed experiments. HC: conducted quantitative genetics analysis. AJ, HC, KH, AW, AK, and BL: performed experiments. AK: performed genotyping and wrote the initial GBLUP script. BL: performed image analysis. HC: wrote the manuscript with contributions from all authors. All authors contributed to the article and approved the submitted version.

## Funding

The authors acknowledge funding from the Biotechnology and Biological Sciences Research Council (BBSRC) BB/M01200X/2 and Innovate UK project 101914.

## Conflict of Interest

The authors declare that the research was conducted in the absence of any commercial or financial relationships that could be construed as a potential conflict of interest.

## Publisher's Note

All claims expressed in this article are solely those of the authors and do not necessarily represent those of their affiliated organizations, or those of the publisher, the editors and the reviewers. Any product that may be evaluated in this article, or claim that may be made by its manufacturer, is not guaranteed or endorsed by the publisher.

## Supplementary Material

Supplementary Figure 1 Manhattan plot of GWAS looking at the association between SNPs and the number of strawberries. 1A to 7D represent the 28 chromosomes of the strawberry genome. The inner Manhattan plot represents class 1 number, followed by class 2 number and total number with the outermost plot representing a marketable number. The pink dotted line represents Bonferroni correction at −log10 p = 7.14, pink points are those which pass the significance threshold. Marker positions are scaled to the Fragaria vesca genome v.4 (Li et al., 2019). The colour-coded key in the outermost circle represents the number of SNPs segregating at each point across the chromosome.

Supplementary Figure 2 Subjective overall texture rating for each strawberry texture type.

Supplementary Figure 3 Average height to width ratio (H:W) for each manually classified strawberry shape category. Desirability in coloured text terms denotes the breeding goals for strawberry shape within the UK. Misc - Miscellaneous undulating misshapen fruit without a clear shape.

Supplementary Table 1 Mean, standard errors (SE), minimum (min) and maximum (max) values for fruit quality traits. Values were provided for each of the families studied. Malling Centenary has been abbreviated to M. Centenary. FA codes denote the names of accessions.

Supplementary Table 2 Visual, textural and organoleptic trait category descriptors of strawberries. Texture type and shape were assessed as discrete ordinal categorical traits and provide context for Texture Rating and Height: Width measures respectively. Texture Type and Shape were not assessed for genetic components.

Supplementary Table 3 Upper and lower bounds of broad-sense heritability (H2) and narrow-sense heritability (h2) for strawberry fruit quality and yield traits across the multi-parental population. Model denotes the BLUEs model fitted per trait where the term DV represents the date of picking and visual recorder specified as random effects, DO represents the date of picking and organoleptic recorder specified as random effects. Variation in date superseded variation in the block. B represents block specified as a random effect D represents the date of picking specified as a random effect. All prediction models were weighted by replicate number. The impact of block, picking date and genome by environment interactions (G × E) on traits; significance values are ANOVA tests comparing mixed models. p values are denoted by stars: ^***^ <0.001, ^**^ <0.01, ^*^ <0.05, <0.01 NS - not significant. Importance denotes the importance of breeding on a scale from 1 (not important) to 9 (highly important). The number of quantitative trait nucleotides (QTN) identified through GWAS after Bonferroni correction. The coefficient of determination (R2) indicates the proportion of variation explained by the combined QTN.

Supplementary Table 4 Mean and SEs for fruit quality traits. Values were provided for each of the blocks studied. NT - Not tested due to plant protection products prohibiting the consumption of fruit.

Supplementary Table 5 QTN associated with strawberry yield and fruit quality traits identified through a GWAS. Bold marker names were associated with multiple traits.

Supplementary Table 6 Mean, SE for fruit yield traits. Values were provided for each of the families studied. Malling Centenary has been abbreviated to M. Centenary. FA codes denote the names of accessions.

## Abbreviations

i35k: Istraw35 Affymetrix chip
GEBV: genomic estimated breeding value
GWAS: genome-wide association study
QTL: quantitative trait locus
QTN: quantitative trait nucleotide
QR: quick response
SNP: single nucleotide polymorphism.
