# Supplementary figures and images for: Genomic Informed Breeding Strategies for Strawberry Yield and Fruit Quality Traits
Source: Front Plant Sci. 2021 Oct 5;12:724847. doi: 10.3389/fpls.2021.724847 (PMC8525896; doi:10.3389/fpls.2021.724847)

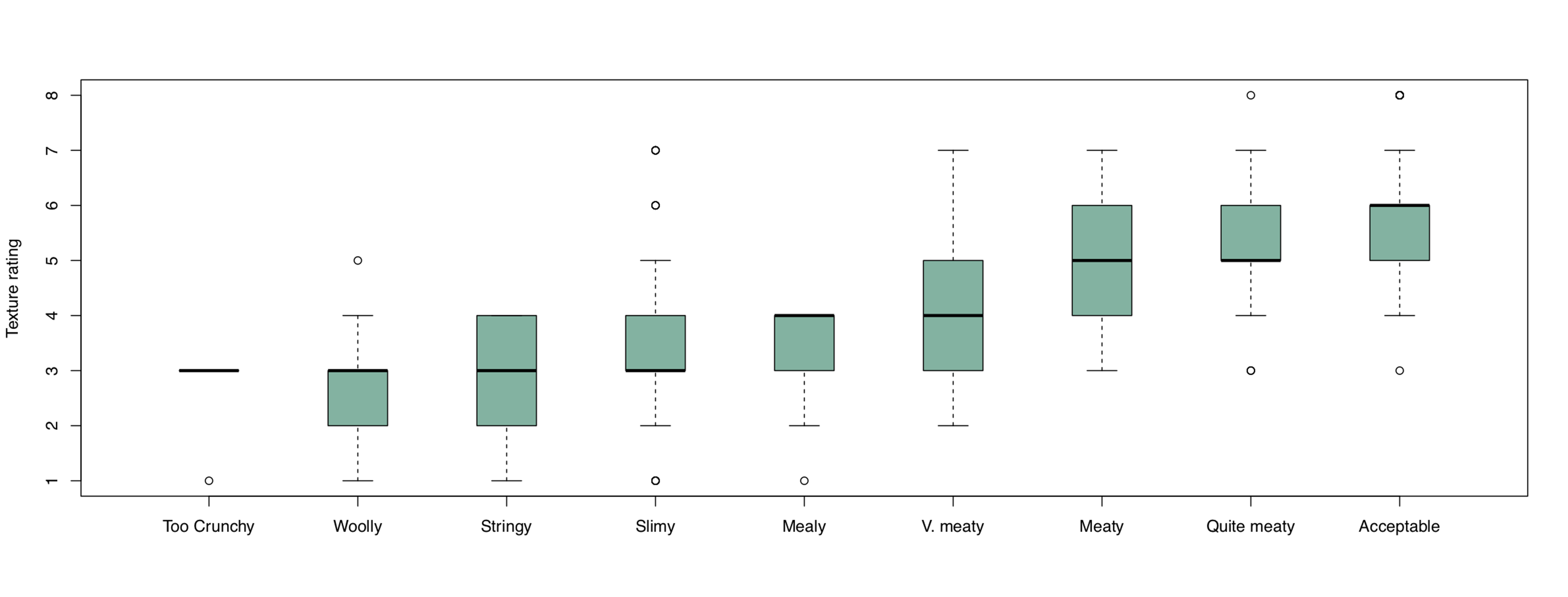

Supplement: Supplementary Figure 2 — Subjective overall texture rating for each strawberry texture type. [file Image_2.tif]

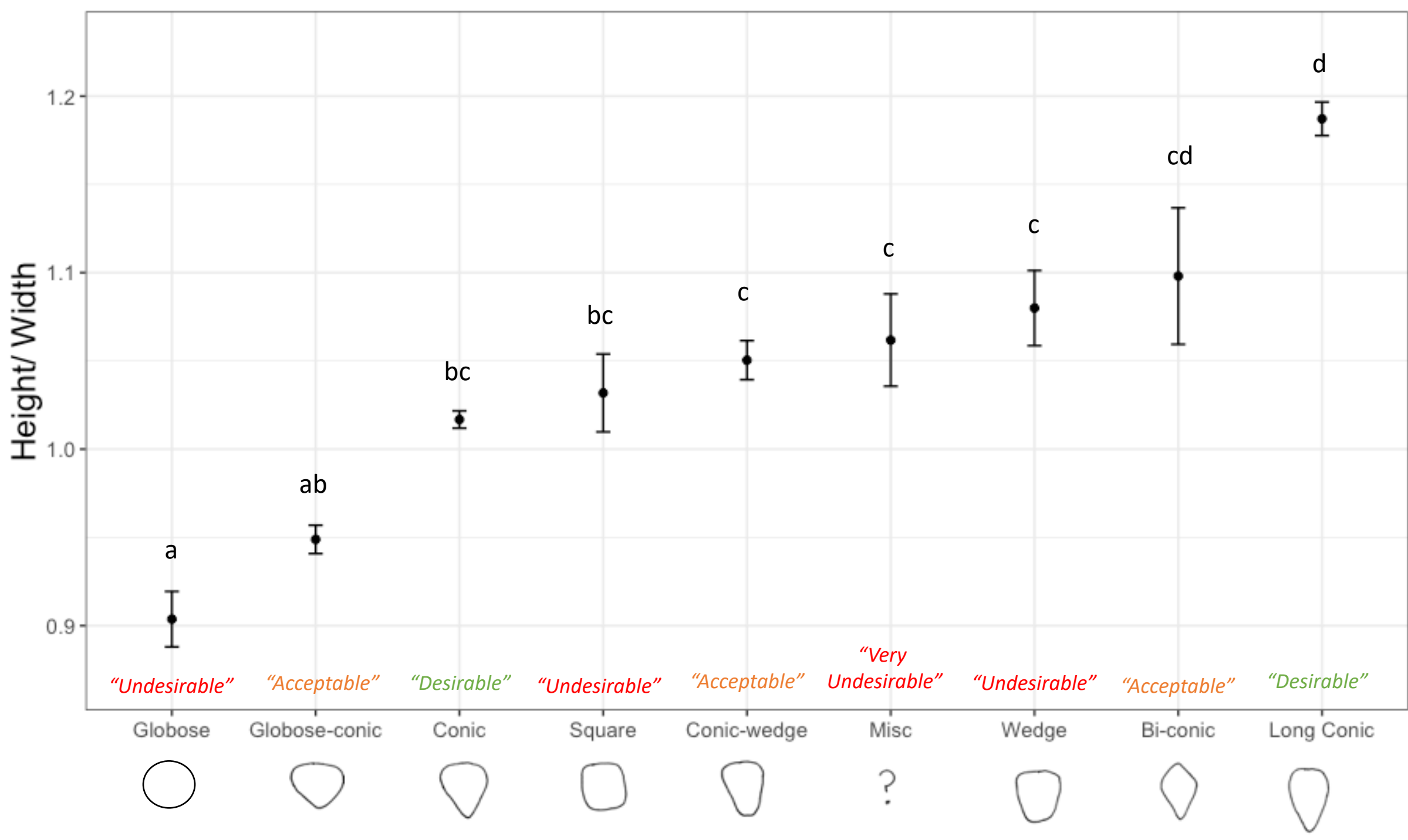

Supplement: Supplementary Figure 3 — Average height to width ratio (H:W) for each manually classified strawberry shape category. Desirability in coloured text terms denotes the breeding goals for strawberry shape within the UK. Misc - Miscellaneous undulating misshapen fruit without a clear shape. [file Image_3.pdf]
